# Isolation and Characterization of Two Bacteriophages Infecting *Bacillus anthracis*: Biological Properties and Genomic Analysis

**DOI:** 10.3390/microorganisms14040777

**Published:** 2026-03-30

**Authors:** Xinyu Qin, Zongti Shao, Binbin Yu, Rongji Cao, Haipeng Zhang, Liyuan Shi, Pan Liu, Shaogui Zi, Jiao Yang, Ying Long, Cong Liu, Siyu Yan, Xiaoxia Yang, Zhengling Zhu, Youhong Zhong, Peng Wang

**Affiliations:** 1 School of Public Health, Dali University, Dali 671000, China; 15378300879@163.com (X.Q.);; 2 Yunnan Provincial Key Laboratory of Natural Epidemic Diseases Prevention and Control Technology, Yunnan Institute of Endemic Diseases Prevention and Control, Dali 671000, China; shaozt_923@163.com (Z.S.);; 3 School of Public Health, Kunming Medical University, Kunming 650500, China

**Keywords:** *Bacillus anthracis* phage, *Bacillus anthracis*, biological characteristics, genomic analysis, Basilis-like phage

## Abstract

Anthrax is a zoonotic infectious disease characterized by high lethality and transmissibility. Its spores are highly stable and can persist in the environment for long periods. Furthermore, the overuse or improper use of antibiotics may contribute to bacterial resistance, complicating anthrax treatment. Phages can efficiently target and lyse *Bacillus anthracis* (*B. anthracis*), significantly reducing pathogen contamination and transmission risks in soil, water, and other environmental media. Compared to traditional chemical disinfectants and antibiotics, phages enable precise pathogen elimination while minimizing ecological disruption. In this study, two phages infecting *B. anthracis*, vB_BanM-JC307 (JC307) and vB_BanS-YL5 (YL5), were isolated and characterized. Both phages belong to the class *Caudoviricetes*. Genome sequencing revealed that JC307 and YL5 have sequence lengths of 148,323 bp and 74,568 bp, respectively. Phylogenetic analysis indicates that JC307 is located in the same evolutionary branch as the Nachito phage of the *Herelleviridae* family, while YL5, although grouped with the Basilisk-like phages, forms an independent branch. As these two phages have been observed to exhibit lytic activity against all nine tested strains of *B. anthracis*, they could serve as auxiliary tools for pathogen diagnosis and assist in ecological management of anthrax-contaminated areas.

## 1. Introduction

Anthrax is a zoonotic infectious disease with high lethality and marked transmissibility [1]. In 1876, the microbiologist Robert Koch demonstrated that anthrax is caused by *Bacillus anthracis* (*B. anthracis*), the first bacterial pathogen confirmed in humans [2]. *B. anthracis* can survive in the environment for decades in the form of spores. Under the induction of mammalian body temperature (37 °C) and physiological signals from the interstitial fluid microenvironment, *Bacillus anthracis* spores can germinate directly in the extracellular space at the site of infection. Once environmental conditions become favorable, these spores germinate and develop into vegetative cells, which may lead to infections in both humans and animals [3]. Naturally occurring human cases are sporadically reported, primarily among individuals exposed to contaminated animal products [4]. Due to its high lethality, low production cost, and odorless nature, *B. anthracis* has been regarded as a critical biological warfare agent [5].

The primary virulence factors of *B. anthracis* are its capsule and anthrax toxin [6]. After invading the host, *B. anthracis* grows and reproduces, forming a capsule that enables it to evade phagocytosis by immune cells such as macrophages, thereby facilitating extensive proliferation in humans or animals [7]. Anthrax toxin consists of protective antigen (PA), edema factor (EF), and lethal factor (LF), which are key factors in the pathogenicity of *B*. *anthracis*. EF and LF are nontoxic outside cells and cannot enter cells on their own. However, when PA binds to cell surface receptors, it mediates the entry of EF or LF into the cell, leading to the formation of edema toxin (EdTx) and lethal toxin (LeTx), respectively, which then exert their toxic effects within the host [6].

Moreover, the misuse or inappropriate use of antibiotics may induce bacterial resistance, thereby complicating anthrax treatment. Compared with antibiotics, phage preparations are widely used in agriculture, animal husbandry, aquaculture, and human disease treatment due to their advantages such as fewer adverse effects, lower likelihood of inducing drug resistance, high safety profile, no residue, and low cost. In recent years, phage cocktail therapy and combined phage-antibiotic treatment have achieved significant efficacy in the management of drug-resistant bacterial infections. Bacteriophage therapy offers a promising alternative, although its application against *B. anthracis* remains at an early stage [8,9,10]. The strong environmental adaptability and persistent survival capacity of *B. anthracis* make natural transmission difficult to control. Conventional disinfection methods also have limitations in eliminating *B. anthracis*, making human anthrax infection a major global public health concern [11]. There is a need to expand the repository of anti-anthrax bacteriophages and explore their genetic diversity. We hypothesize that the unique environment of Yunnan, a biodiversity-rich region of China, may serve as a source of novel and highly effective bacteriophages targeting *B. anthracis*.

In China, research on *B. anthracis* phages has been relatively limited, with only three strains isolated and characterized to date—a number far from sufficient to support fundamental studies, diagnostic development, or ecological control strategies. In this study, two novel *B. anthracis* phages, vB_BanM-JC307 (JC307) and vB_BanS-YL5 (YL5), were isolated from soil samples collected in Yunnan Province. Through comprehensive analysis of their biological and genomic features, this study contributes to expanding the national phage repository and provides a foundation for the development of rapid diagnostic tools, environmental decontamination agents, and phage-based ecological interventions. These efforts are of practical significance for strengthening the capacity for anthrax prevention and control, as well as enhancing preparedness for public health emergencies in Yunnan Province and beyond.

## 2. Materials and Methods

The host range tests were conducted in a biosafety level 3 laboratory (BSL-3), while the remaining experiments were completed in a biosafety level 2 laboratory (BSL-2). All the strains used in the experiment were obtained from the Local Disease Control Institute of Yunnan Province.

### 2.1. Bacterial Strains and Culture Conditions

The *B. anthracis* A16R vaccine strain was used as the host for phage amplification. Bacteria were cultured in Luria–Bertani (LB) medium, either on solid agar plates containing 1.5% agar or in liquid broth. Cultures were incubated at 37 °C with shaking at 220 rpm.

### 2.2. Isolation and Identification of Bacteriophages

Jianchuan County (26°30′28.8″ N, 100°12′8.0″ E; altitude: 3199 m) and Yulong County (26°44′30.9″ N, 100°5′14.6″ E; altitude: 2827 m) in Yunnan Province were previously identified as anthrax-endemic areas and have also experienced historical plague outbreaks. Given that rodents are explicitly recognized as vectors for anthrax transmission, soil samples were collected from rodent burrows in these regions using a purposive sampling strategy. A total of 639 samples were obtained at a depth of 10 cm. Notably, the two samples from which phages JC307 and YL5 were isolated consisted of soggy soil.

The isolation procedure was as follows: 10 g of fresh soil sample was thoroughly mixed with 10 mL of SM buffer (50 mM Tris-HCl (pH 7.5), 100 mM NaCl, 8 mM MgSO_4_, 0.01% gelatin) (Sinopharm Chemical Reagent, Shanghai, China) and centrifuged at 5000× *g* for 10 min. The supernatant was filtered through a 0.22 μm membrane filter (Merck Millipore, Darmstadt, Germany). Then, 500 μL of the filtrate was mixed with 200 μL of host bacterium (*B. anthracis* vaccine strain A16R) in 7 mL of LB broth and incubated overnight at 37 °C. After incubation, the culture was centrifuged (3000× *g*), and the supernatant was again filtered through a 0.22 μm filter for further use. For plaque purification, 200 μL of filtered lysate was mixed with 300 μL of exponentially growing *B. anthracis* A16R culture, immediately added to 7 mL of molten LB soft agar (approximately 45 °C), and poured onto solidified LB agar plates. Plates were incubated at 37 °C for 12 h to allow plaque formation. A single, well-isolated clear plaque was picked using a sterile pipette tip and transferred into 6 mL of LB broth containing *B. anthracis* A16R bacteria. This mixture was incubated at 37 °C with shaking at 220 rpm for 8–9 h. The lysate was filtered through a 0.22 μm filter, serially diluted, and further purified using the double-layer agar method. This purification step was repeated at least three times to ensure phage homogeneity. Purified phage stocks were amplified in *B. anthracis* A16R host cultures and stored in SM buffer at 4 °C for long-term preservation. These preparations were used in subsequent analyses.

Preparation methods for various types of LB culture media (Table 1).

The volume of purified water depends on the amount of culture medium needed each time, and all prepared culture medium must be sterilized at 121 °C for 30 min before use.

### 2.3. Transmission Electron Microscopy

Purified phage suspensions were applied to 400-mesh carbon-coated copper grids for 15 min, negatively stained with 2% phosphotungstic acid (PTA, pH 6.5) for 30 s, and examined by transmission electron microscopy [12]. Phage dimensions represent the mean values from four particles (HT7700 Exalens) (Hitachi, Tokyo, Japan).

### 2.4. Optimal Multiplicity of Infection Assay and One-Step Growth

The optimal multiplicity of infection (MOI) [13] was determined by mixing phage suspensions with the *B. anthracis* A16R host bacterium at varying ratios, and incubating the mixtures at 37 °C for 6 h. Phage titers for each ratio were quantified by serial dilution and spot titration. The ratio yielding the highest titer was identified as the optimal MOI.

In subsequent experiments, phage lysate and *B. anthracis* A16R culture were mixed at a 1:1 ratio and adsorbed for 30 min at 37 °C with shaking (220 rpm). Time-course sampling was initiated at t = 0 min and continued at 5 min intervals for the first 20 min and 10 min intervals thereafter until 110 min post-infection. At each time point, phage titers were measured using the double dilution method and spot test. Plaques were counted and recorded to construct the one-step growth curve [14].

### 2.5. Sensitivity of Phages to Temperature and PH

Phage thermal stability was evaluated across seven temperature conditions (4 °C, 21 °C, 28 °C, 37 °C, 50 °C, 60 °C, and 70 °C). For each temperature condition, 1 mL aliquots of standardized phage lysate were incubated for 60 min. After incubation, 100 μL samples were serially diluted and plated using the double-layer LB agar method.

To assess pH tolerance, phage sensitivity was examined across a pH gradient (1–13) prepared in PBS buffer, adjusted with NaOH or HCl. Aliquots (100 μL) of high-titer phage lysates (>10^8^ PFU/mL) were suspended in 1 mL of each pH solution and incubated at room temperature for 1 h. Treated suspensions were serially diluted and plated on double-layer LB agar to evaluate viability.

### 2.6. Determination of the Host Spectrum

A total of nine *B. anthracis* strains, one *B. cereus* strain were selected for host analysis. Equal volumes (100 μL) of undiluted phage solution (titer > 10^8^ PFU/mL) were applied onto top-layer agar plates containing 200 μL of bacterial cultures. Plates were incubated at 37 °C for 12 h, and plaque formation was recorded.

### 2.7. Genomic DNA Extraction and Sequencing

Phage genomic DNA was extracted using the λ Phage Genomic DNA Extraction Kit (Abigail Crop, Beijing, China) according to the manufacturer’s instructions. DNA samples were randomly fragmented with a Covaris M220 acoustic focusing device (Covaris, Woburn, MA, USA) to achieve suitable fragment lengths. Sequencing libraries were prepared and sequenced using the Illumina NovaSeq 6000 platform with paired-end sequencing. Raw sequencing data underwent quality control using Fastp (v0.20.0) [15]. Host, bacterial, and rRNA sequences were removed by comparing reads against corresponding NCBI databases using BBmap software v38.51 [16]. Clean reads were assembled de novo, and contigs were identified via BLAST (v2.10.0+) against the virus-NT database. Completeness and accuracy of assemblies were evaluated.

### 2.8. Genome Analysis

Similar phage sequences were identified through BLASTn [17] searches against the GenBank database. Genome annotation was performed automatically via the RAST online platform [18], and resulting annotations were manually curated using BLASTP [19] from NCBI. The genomic map was generated with SnapGene (v8.0.0). Phylogenetic analysis and multiple sequence alignments were conducted using MEGA11 [20] and ClustalW [21], and results were visualized with Chiplot. Synteny alignments were visualized using Easyfig (v2.2.5) [22] and refined with Adobe Photoshop 2022 (PS). Average Nucleotide Identity (ANI) values between phage genomes were calculated using BioAider [23], and a heatmap was generated by importing a square matrix into Chiplot via Excel. Conserved domains and active sites in phage tail structural proteins were predicted using the Conserved Domain Database (CDD) [24] from NCBI and visualized using Chiplot. Sequence alignments were visualized and refined with GeneDoc.

## 3. Results

A total of 13 *B. anthracis* phages were isolated from the 639 soil samples, including the myovirus JC307, the sole representative of its morphotype, and the ultralong-tailed siphovirus YL5, both of which were subjected to further analysis.

### 3.1. Phage Host Range and Morphology

Plaques formed by JC307 and YL5 exhibited distinct morphological characteristics. JC307 produced large, transparent plaques (2–3 mm diameter), whereas YL5 formed smaller, transparent plaques (approximately 1 mm diameter).

**Figure 1:**
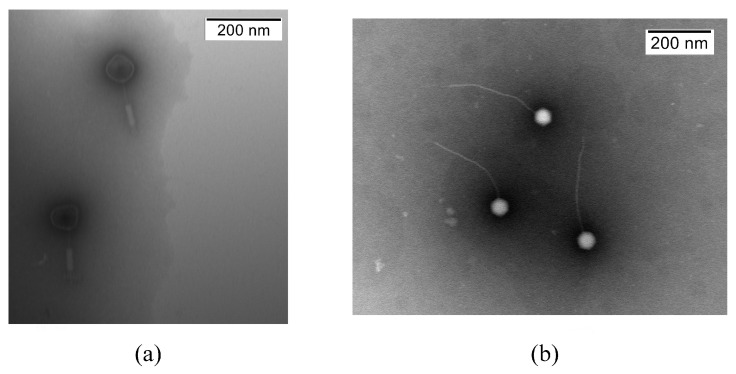
Transmission electron micrographs of (**a**) JC307, (**b**) YL5. For TEM, the phages were stained with 2% phosphotungstic acid.

JC307 possessed a typical icosahedral head (55 ± 5 nm) and a long, contractile tail (180 ± 5 nm), consistent with myovirus morphology. In contrast, YL5 had a similar-sized icosahedral head (54 ± 5 nm) but featured a significantly longer, non-contractile tail (310 ± 10 nm), characteristic of phages from siphovirus morphology. Electron microscopy confirmed that both phages belonged to the class *Caudoviricetes* (Figure 1). Host ranges of JC307 and YL5 are summarized in Table 2. JC307 fully lyses the *B. anthracis* vaccine strain A16R and all other tested *B. anthracis* strains; YL5 fully lyses only *B.anthracis* A16R, produces only partial lysis (turbid plaques) on other *B. anthracis* strains, but is capable of lysing *Bacillus cereus* BC248.

### 3.2. Sensitivity of Phages to Temperature and pH

**Figure 2:**
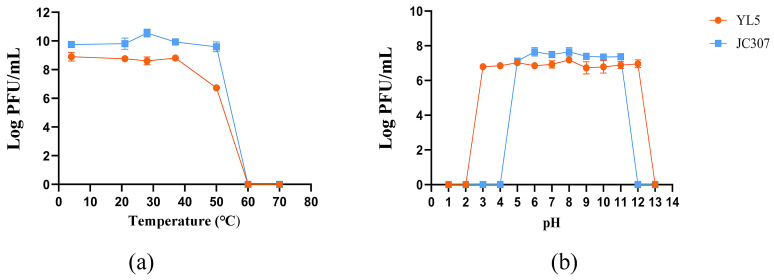
(**a**) Thermal stability of phage JC307 and YL5. (**b**) Survivability of phages JC307 and YL5 upon incubation at different pHs. Error bars represent the SD of the mean from three replicate experiments.

YL5 maintained stable titers between 4 °C and 37 °C. At 50 °C, its titer rapidly decreased, but residual activity remained. At 60 °C, YL5 was completely inactivated. In contrast, JC307 remained stable within a temperature range of 4 °C to 50 °C but lost all activity at 60 °C (Figure 2a).

Regarding pH stability, JC307 remained active within a pH range of 5–11, with complete inactivation occurring at pH < 5 or >11. YL5 demonstrated greater pH tolerance, retaining activity between pH 3–12 and losing all viability at pH < 3 or >12 (Figure 2b).

### 3.3. Optimal Multiplicity of Infection Assay and One-Step Growth

**Figure 3:**
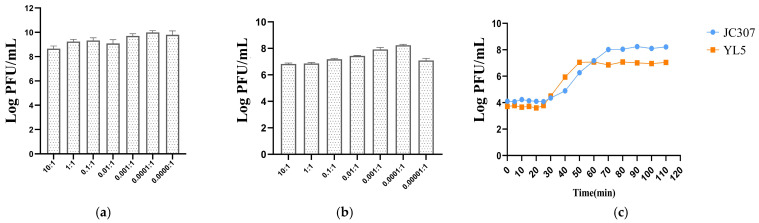
(**a**) Optimal multiplicity of infection of JC307. (**b**) Optimal multiplicity of infection of YL5. (**c**) One-step growth curve. Error bars represent the SD of the mean from three replicate experiments.

The optimal multiplicity of infection (MOI) for JC307 and YL5 was 0.0001 (Figure 3a,b). However, their one-step growth curves differed notably. JC307 had a latent period of approximately 30 min, followed by a rapid increase in phage titer between 30 min and 70 min, indicating a lysis period of 40 min. Titers stabilized after 70 min. Titers stabilized after 70 min. In contrast, YL5 exhibited a shorter latent period (approximately 25 min) and a quicker burst period, with a rapid titer increase between 25 min and 50 min, indicating a lysis period of 25 min. These differences indicated that YL5 possesses a faster replication cycle compared to JC307 (Figure 3c).

### 3.4. General Characteristics of Phage Genome Sequences

The genome lengths of JC307 and YL5 are 148,323 bp and 74,568 bp, respectively. Through RAST annotation, JC307 was found to encode 229 proteins, while YL5 encodes 107 proteins. Neither tRNA nor rRNA genes were identified. Analysis using the CARD and VFDB databases [25] detected no antibiotic resistance genes or virulence factors. The genomes contain eight structural and functional modules: structural proteins, tail structures, DNA packaging, lysis, lysogeny, DNA replication and transcription, auxiliary metabolic genes (AMGs), and unknown-function modules.

**Figure 4:**
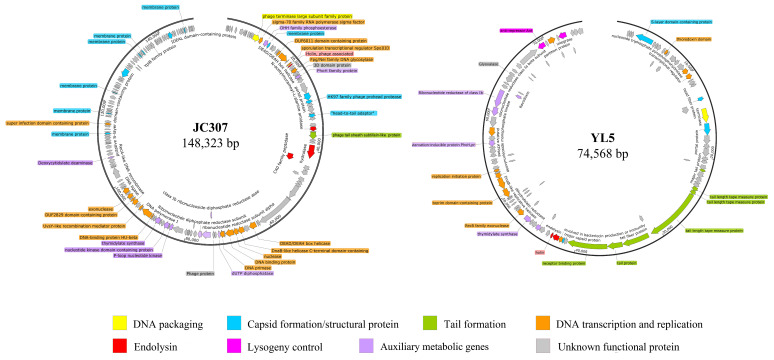
Comparison of schematic genomic maps of phage JC307 and YL5. Genes are color-coded based on their predicted functions.

The bacteriophage DNA packaging module includes the terminase complex, responsible for cutting newly synthesized DNA and packaging it into the capsid [26,27]. Capsid proteins and tail modules together form the head, neck, and tail structures. In JC307, a perforin-lysozyme binary lysis system was identified, comprising holin (ORF33), endolysin (ORF53), hydrolase (ORF59), and C40 family peptidase (ORF60). JC307 lacks a lysogenic module, indicating it is a strictly lytic phage. In contrast, YL5 encodes holin (ORF48), endolysin (ORF47), as well as an integrase (ORF104) that mediates phage integration into the host genome [28]. Additionally, YL5 contains an anti-repressor protein (Anti-repressor Ant, ORF99) (Figure 4).

JC307 and YL5 exhibit low genomic similarity to previously isolated anthrax bacteriophages in China, including AP631 [29] (25.43%; 35.07%), A16R1 [30] (25.38%; 35.01%), and A16R4 [30] (22.45%; 28.89%). Through BLASTn analysis, JC307 shares high similarity (coverage: 96%; identity: 97.0%) with Bacillus phage Nachito. In contrast, YL5 shares only moderate similarity (coverage: 67%; identity: 88.31%) with its closest relative, Bacillus phage Basilisk. Given this level of divergence [31], YL5 is considered a novel anthrax bacteriophage. The genomic characteristics of JC307 and YL5 are summarized in Table 3.

Auxiliary metabolic genes (AMGs) potentially facilitating phage replication were identified within DNA and nucleotide metabolism modules. The identified AMGs are related to central carbon metabolism, nitrogen metabolism, phosphorus and sulfur cycling, nucleotide metabolism, and oxidative stress responses [32,33]. During prolonged co-evolution with hosts, bacteriophages frequently acquire host DNA fragments to enhance survival, evade host immune defenses, or improve ecological fitness [34]. Comparative genomic analyses of anthrax phages isolated in China revealed notable differences in AMG content. JC307 and YL5 both harbor AMGs linked to nucleotide metabolism and phosphorus cycling. AP631 and A16R1 lack these genes entirely, whereas A16R4 encodes only the nucleoside triphosphate pyrophosphohydrolase (MazG). JC307 and YL5 both encode ribonucleotide reductases (RNRs), albeit annotated differently: JC307 encodes homologs of NrdI (gp84), NrdE (gp85), and NrdF (gp86), whereas YL5 encodes flavodoxin (gp87), class Ib RNR (gp86), and ribonucleotide-diphosphate reductase subunit beta (gp85). Both phages encode thymidylate synthase (thyX), crucial for pyrimidine biosynthesis [35]. JC307 also encodes dUTP diphosphatase and deoxycytidylate deaminase, while YL5 encodes MazG and dihydrofolate reductase (DHFR). Both phages contain homologs of the PhoH protein, indicating a potential conserved mechanism for environmental adaptation [36]. AMG categories in our phages as well as other anthrax phages isolated in China (AP631, A16R1, A16R4) are shown in Table 4.

### 3.5. Phylogenetic Analysis

NCBI searches identified only phage Nachito as closely related to JC307 (>70% similarity), whereas YL5 displayed five additional genomes exceeding 70% similarity (phages v_B-Bak1, v_B-Bak6, v_B-Bak10, pW4, and PBC4) beyond phage Basilisk. Phylogenetic analyses incorporated previously characterized anthrax phages, including phages from *Wbetavirus* genus (Wbeta, Gamma, Fah, J5a, F16Ba, z1a), phage AP50 (*Betatectivirus*), phage crookii (*Wphvirus*), and three Chinese anthrax phages (AP631, A16R1, A16R4).

**Figure 5:**
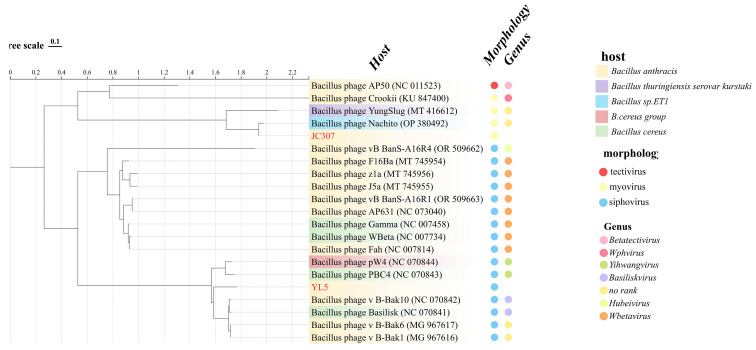
Phylogenetic analysis of JC307 and YL5 as well as other anthrax bacteriophages based on the whole genome sequences. The gradient color module in the figure represents the host of the bacteriophage. The two columns on the right, respectively, indicate the morphology of the bacteriophages and those that have been classified into specific genera.

According to the ICTV classification system, the analyzed bacteriophages belong to eight genera, with JC307 and YL5 assigned to different branches. JC307 grouped within *Herellevirus* family, forming a monophyletic cluster with phage Nachito, indicating a close evolutionary relationship. YL5, although closely related evolutionarily to phage Basilisk, formed an independent branch, suggesting it represents a distinct genetic lineage (Figure 5).

### 3.6. Comparative Genomic Analysis

**Figure 6:**
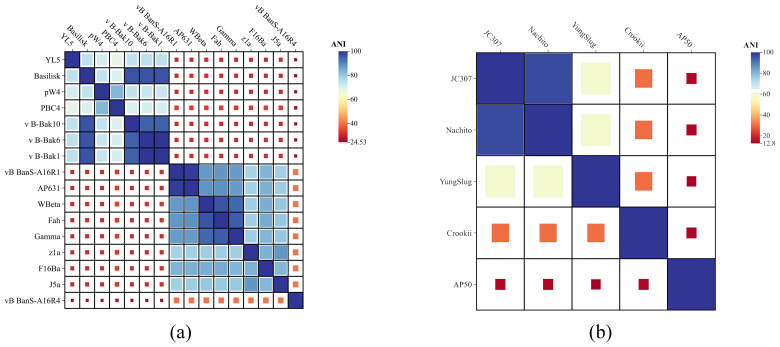
(**a**) The homology and average nucleotide identity (ANI) of YL5. (**b**) The homology and average nucleotide identity (ANI) of JC307.

Comparative genomic analyses of JC307, YL5, and other anthrax-associated phages were performed using BioAider. The ANI showed YL5 clustering with Basilisk-like phages (BLPs) at similarity >70% but <95%. It exhibited low similarity to members of genus Wbetavirus, which formed a distinct phylogenetic cluster (Figure 6a). JC307 demonstrated high similarity (>95% ANI) with phage Nachito but only 58.78% ANI with its congeneric phage YungSlug, indicating substantial intrageneric divergence (Figure 6b).

**Figure 7:**
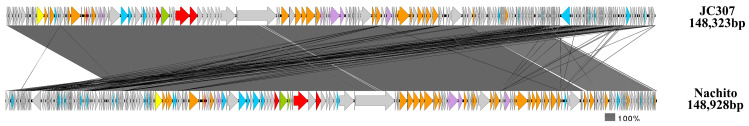
Genome collinearity of phages YL5, Bailisk, v_B-Bak10, v_B-Bak1, pW4 and PBC4. Predicted functional proteins are represented in different colors, with arrows indicating gene length and transcription orientation. Shading beneath the genomes represents sequence similarity, with the color intensity reflecting the degree of similarity.

Syntenic collinearity analyses compared phages YL5 and JC307 with other phages showing >70% sequence similarity. Among the compared phages, YL5 possessed the smallest genome. Within its structural module, a unique S-layer domain-containing protein and a major capsid protein were identified. The S-layer domain-containing protein showed 100% coverage and 77.18% similarity to Bacillus phage BC01, while the major capsid protein exhibited 91% coverage and 47.76% similarity to Bacillus phage BCPST. Other structural modules demonstrated collinearity with phages Basilisk and pW4. The initial tail structural protein regions of YL5 partially aligned with BLPs, but its receptor-binding protein (RBP) and terminal tail protein showed low similarity. The closest RBP homolog identified was in Bacillus phage BSG01 (100% coverage, 58.33% similarity). Functional modules, packaging, nucleotide metabolism, lysis, and lysogeny, showed clear similarity with corresponding modules in BLPs (Figure 7).

**Figure 8:**
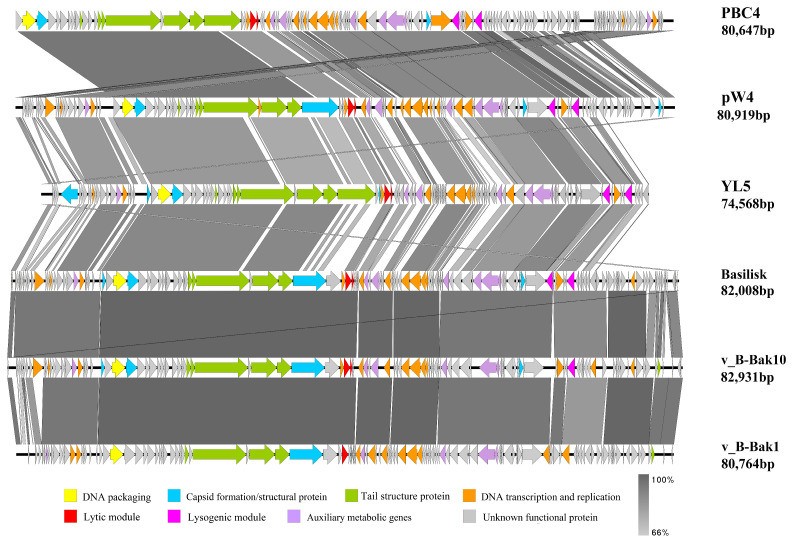
Genome collinearity of phages JC307 and Nachito. Predicted functional proteins are represented in different colors, with arrows indicating gene length and transcription orientation. Shading beneath the genomes represents sequence similarity, with the color intensity reflecting the degree of similarity.

Due to the high similarity between the bacteriophage JC307 and the bacteriophages Nachito (>95% sequence similarity). JC307 and Nachito displayed significant sequence conservation across functional modules, including capsid assembly, tail structure, and nucleotide metabolism. However, genomic inversions were observed in some structural module positions (Figure 8).

### 3.7. Sequence Analysis of Phage Tail Structural Proteins

The bacteriophage tape measure protein (TMP) determines the tail length and facilitates genome translocation into the host [37,38]. The TMP gene (gp39) in phage YL5 is the largest within its genome (6565 amino acids). This TMP exhibits 83.46% similarity to Basilisk gp118, 85.85% similarity to PBC4 TMP, and highest similarity (85.91%) to BCPST TMP. Domain prediction analysis revealed multiple conserved domains in YL5 GP39: an N-terminal phage-associated domain (YqbO-like superfamily) overlapping with a conserved tape measure domain (TP901), a LysM-containing MepM/NlpD endopeptidase domain overlapping with a peptidase M23 domain (NlpD), and a C-terminal domain identified as Rad50 ATPase, involved in DNA double-strand break repair [39].

**Figure 9:**
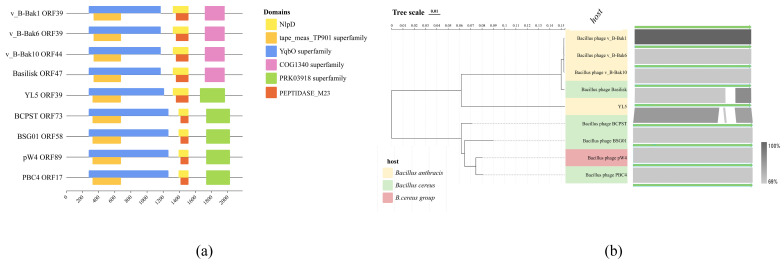
(**a**) The prediction results of the conserved domains of YL5 tape measure protein gp39 and Basilisk-like phages TMP. (**b**) The tape measure protein phylogenetic tree of YL5 and the collinearity analysis.

Comparative domain analysis of YL5 TMP and related *Bacillus*-specific phages indicated broad conservation of the TP901-like domain among BLPs [40]. The predicted NlpD domain contains a peptidase M23 domain, a zinc metallopeptidase within the Gly-Gly endopeptidase family. This domain likely facilitates binding and cleavage of host peptidoglycan [41]. The predicted Rad50 ATPase domain at the C-terminus of YL5 TMP resembles the domain in phage PBC4 but differs from that of Basilisk (Figure 9a).

Phylogenetic analysis revealed that TMP of YL5 gp39 forms a distinct monophyletic branch, indicating genetic diversity. In synteny analyses, TMPs from phages v_B-Bak1 gp39 and v_B-Bak6 gp39 showed high sequence similarity. Alignments of YL5 gp39 with Basilisk gp47 and BCPST GP73 indicated regions of low similarity. However, YL5 displayed the highest overall similarity (85.91%) with phage BCPST (Figure 9b).

**Figure 10:**
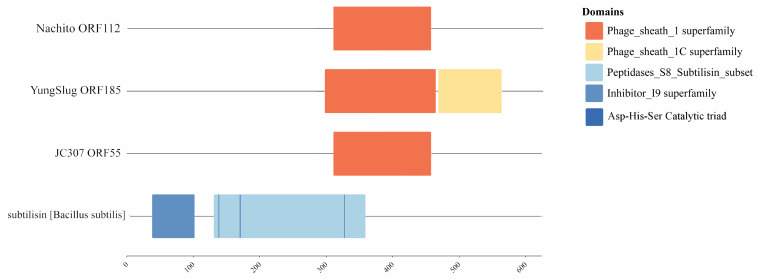
The prediction results of the conserved domains of JC307 gp55 and *Herellevirus* phage tail protein.

The tail structural module of JC307 encodes only one tail sheath protein (gp55) containing a subtilase-like domain (residues 311–458). Domain comparisons between gp55 and homologous tail proteins revealed that although JC307 gp55 contains this subtilase-like domain, it lacks the catalytic triad (Asp-His-Ser), suggesting no proteolytic activity [42]. Thus, the domain likely remains as a structural scaffold essential for tail assembly and stability, rather than functioning enzymatically (Figure 10).

### 3.8. Bioinformatics Analysis of Lyase

**Figure 11:**
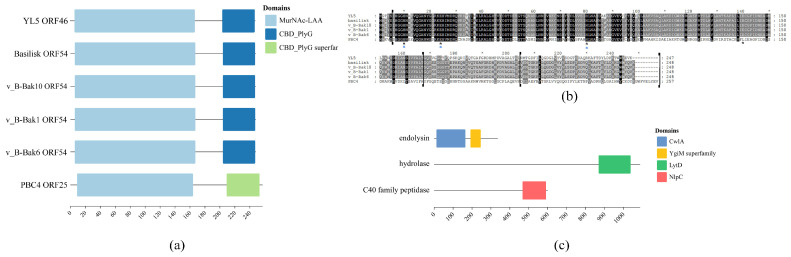
(**a**) The prediction results of the conserved domain of YL5 autolysin gp48 and similar to the bacterial lysis enzyme of Basilisk bacteriophage. (**b**) The sequence alignment results of YL5 gp48 and BLPs hydrolase. * Indicates the active site of the enzyme; * Indicates the active site of zinc ions (**c**) The prediction results of the conserved domains of the three hydrolase enzymes in JC307.

Gram-positive autolysins typically have modular structures with an N-terminal catalytic domain and a C-terminal cell wall-binding domain (CBD) [43]. The autolysin from YL5 contains an N-terminal MurNAc-LAA amidase domain (residues 6–166) and a C-terminal PlyG CBD (residues 203–246) (Figure 11a). This lyase closely resembles those encoded by BLPs. Conserved domain analysis identified four active-site residues (His-10, Glu-24, His-80, Glu-140) forming a catalytic His-Glu-His-Glu tetrad. Three residues (His-10, Glu-24, His-80) are zinc-binding sites essential for hydrolase activity. However, the C-terminal CBD of the YL5 autolysin, although classified as CBD_PlyG, differs significantly in sequence from those in Basilisk and Bak10, possibly conferring unique host-specific recognition (Figure 11b).

Phage JC307 encodes three predicted lytic enzymes: an endolysin, a hydrolase, and a C40 family peptidase. The endolysin contains an N-terminal conserved N-acetylmuramoyl-L-alanine amidase domain (CwlA) that specifically hydrolyzes the MurNAc-L-Ala amide bond. Its C-terminal domain (YgiM) possesses an SH3 fold and may be involved in substrate recognition [44]. The hydrolase contains a predicted LytD domain, a β-N-acetylglucosaminidase cleaving glycosidic bonds between GlcNAc-MurNAc residues, inducing bacterial autolysis [45]. The C40 family peptidase carries an NlpC domain with cysteine peptidase activity, targeting peptide cross-links in peptidoglycan [46]. These enzymes act synergistically: the hydrolase initially disrupts glycan backbones, the endolysin cleaves amide bonds, and the C40 peptidase degrades peptide cross-links, causing complete cell-wall destruction (Figure 11c).

## 4. Discussion

In this study, two bacteriophages infecting *B. anthracis*, vB_BanM-JC307 and vB_BanS-YL5, were isolated and characterized. Electron microscopy detection revealed that both viruses belong to the *Caudoviricetes* class of tailed bacteriophages. JC307 formed a new branch with phage Nachito. Although YL5 is similar to BLPs, it formed an independent evolutionary branch, indicating significant genetic differentiation during evolution.

Phage JC307 exhibited high genomic homology with phage Nachito in the database, with a sequence similarity of 97%. Despite the high genomic similarity, significant differences existed in their host ranges. According to NCBI records, the host of Nachito is *Bacillus* sp. ET1, a bacterium from soil. However, JC307 effectively lysed the strict pathogen, *B. anthracis*. The lysing systems encoded by the two phages were compared. Analysis showed that JC307 and Nachito both encode three lysins, among which the endolysin and hydrolase are highly conserved (similarities of 98.21% and 99.45%, respectively). The main difference lies in the third lysin: JC307 encodes a C40 family peptidase, while Nachito encodes a tail-associated lysozyme. Sequence alignment revealed substantial differences between these two proteins. It is thus speculated that this substitution in the lysin module, particularly the transformation from a tail-associated lysozyme to a C40 family peptidase, might explain JC307’s ability to infect *B. anthracis* and its different host range compared with Nachito.

Phages Basilisk, v_B-Bak1, v_B-Bak10, v_B-Bak6, and PBC4 are referred to as “Basilisk-like phages, BLPs”. YL5 clustered with BLPs and showed the highest similarity to Basilisk, suggesting a common ancestral origin. However, detailed collinearity analysis demonstrated extensive genomic differences with generally low sequence similarity among predicted proteins. This indicates that YL5 underwent significant independent evolution after diverging from BLPs, accumulating numerous genetic variations. Therefore, our data strongly support YL5 as a distinct branch within the BLP lineage.

YL5 has an exceptionally long tail structure (approximately 310 nm), longer than that of v_B-Bak10 (200 nm) but shorter than that of Basilisk (420 nm) and v_B-Bak1 and v_B-Bak6 (357 nm). The long tail morphology of phage YL5 is primarily determined by the tape measure protein (TMP). Collinearity comparison of the TMP of BLPs and YL5 (gp39) showed very low similarity, with a region of zero similarity between YL5 gp39 and Basilisk gp47. The highest similarity (85.91%) of YL5 gp39 was with the TMP of phage BCPST (gp73), but a region of zero similarity remained. Additionally, the conserved domain encoded at the C-terminal of YL5 gp39 (PRK03918) is significantly different from Basilisk gp47 (COG1340). However, it matches the conserved domain of BCPST gp73. After the M_23 peptidase conserved domain, BCPST gp73 contains an insertion of 61 amino acids. YL5 also encodes a RBP (gp43). BLAST comparison showed the most similar protein was from bacteriophage BSG01, with 100% coverage but only 58.33% similarity. This indicates a common ancestral origin but substantial sequence divergence during evolution. Since the RBP determines host specificity [47], the significant differences between YL5 and BSG01 RBPs likely result directly in their distinct host ranges.

Phages can influence the composition and metabolism of microorganisms through lytic or non-lytic infection [48]. Certain phages enhance or alter specific metabolic processes of the host by expressing AMGs during infection. Thus, AMGs of phages may enhance the metabolic capacity of host cells and provide adaptive advantages to bacteriophages under specific environmental or nutritional conditions [49]. In the nucleic acid metabolism module, both JC307 and YL5 encode a complete set of RNR genes (NrdI-NrdE-NrdF) [50]. Generally, RNR genes are common in lytic bacteriophages but rare in lysogenic bacteriophages. Therefore, RNR genes are considered closely associated with a lytic lifestyle and are crucial for rapid replication [51]. JC307 also encodes deoxycytidine deaminase and thymidylate synthase, forming an independent dTMP synthesis pathway. This enables JC307 to efficiently and specifically produce dTTP and reduces its dependence on the host’s thymidylate pool [52]. Additionally, the dUTPase encoded by JC307 plays a key role in maintaining genomic integrity by regulating the intracellular dUTP level. This effectively prevents the misincorporation of dUTP into newly synthesized DNA, ensuring accurate replication [53].

Besides encoding thymidylate synthase, YL5 also encodes DHFR. Acid-base tolerance experiments indicated that YL5 can survive at pH 12. Usually, alkaline environments damage the structural stability of nucleic acids and proteins and severely hinder the basic metabolism of microorganisms, inhibiting bacteriophage survival and replication [54]. However, YL5 carries DHFR, catalyzing the conversion of dihydrofolate (DHF) to tetrahydrofolate (THF). As a derivative of vitamin B9, THF is essential for folate-dependent one-carbon metabolism, supporting the biosynthesis of thymidine, purines, and other metabolites [55,56]. Therefore, YL5 likely survives alkaline conditions not due to structural resistance, but by expressing its own DHFR. This expression actively maintains or enhances the host cell’s THF pool, ensuring efficient proliferation under adverse conditions. In addition to AMGs related to nucleotide metabolism, the genomes of JC307 and YL5 also encode the phosphate stress gene phoH. PhoH may enhance the host’s phosphorus acquisition and utilization efficiency, thus supporting bacteriophage replication. Notably, putative phoH homologs are rare in non-marine bacteriophages [36].

As a strictly lytic bacteriophage, JC307 offers significant advantages in clinical applications due to its replication cycle, which lacks a lysogenic stage. This mechanism eliminates the risk of horizontal gene transfer mediated by lysogenic conversion or generalized transduction, ensuring therapeutic safety. In contrast, YL5, as a novel temperate bacteriophage, possesses both lytic and lysogenic life cycles. This flexible life strategy enables it to occupy a unique ecological niche in complex soil microbial communities, ensuring its survival and spread. Thus, YL5 is a promising candidate for the development of long-term bacteriophage-based biological control strategies.

## 5. Conclusions

This study isolated and characterized two novel *B. anthracis* bacteriophages, vB_BanM-JC307 and vB_BanS-YL5, both belonging to the order *Caudoviricetes*. Genomic and phylogenetic analyses revealed that JC307 exhibits high homology with the *Herelleviridae* phage Nachito, whereas YL5 constitutes an independent branch within the Basilisk-like phages (BLPs) lineage. Various auxiliary metabolic genes (AMGs) were identified in the genomes of both phages. JC307 and YL5 serve as candidate resources for the specific lysis of *B. anthracis*, offering new potential tools for the auxiliary diagnosis and ecological control of anthrax.

## Figures and Tables

**Table 1:** Preparation Method for LB (Luria–Bertani) Culture Medium (Using 400 mL as an Example).

Nutrients (g)	LB Agar	LB Soft Agar	LB Broth
Agar (OXIOD, Basingstoke, UK)	6.0	1.6	-
Yeast (OXIOD, UK)	4.0	4.0	4.0
Peptone (OXIOD, UK)	2.0	2.0	2.0
NaCl (XiLong Scientific, Guangzhou, China)	3.6	3.6	3.6
Na_2_HPO_4_ (XiLong Scientific, China)	1.0	1.0	1.0

**Table 2:** Host spectra of JC307 and YL5.

Species	Strains	JC307			YL5		
		37 °C	28 °C	21 °C	37 °C	28 °C	21 °C
*Baciillus anthracis*	A16R						
	Huize						
	Yanglin						
	Midu						
	Kunming-LTM						
	Kunming-MZK						
	K1						
	K2						
	Kunming-FY						
*Baciillus cerese*	BC248						

Annotation: Green: Completely lysed/transparent bacterial plaque; Yellow: Incomplete lysation/turbid bacterial plaque; White: No lysis phenomenon.

**Table 3:** General characteristics of the genomes of two bacteriophages.

Phage	Genome Size	%GC	Number of Predicted Genes	Closest Relative (GenBank Acc. No.)	% Identity
JC307	148,323 bp	33.15%	229	Bacillus phage Nachito (OP_380492)	97%
YL5	74,568 bp	34.05%	107	Bacillus phage Basilisk (NC_070841)	88.31%

**Table 4:** The categories of auxiliary metabolic genes.

Function and Protein	Gene Product of the Following Phage				
	JC307	YL5	A16R1	A16R4	AP631
nucleoside triphosphate pyrophosphohydrolase	\	gp16	gp4	\	\
PhoH family protein	gp39	gp81	\	\	\
dUTP diphosphatase	gp80	\	\	\	\
class Ib ribonucleoside-diphosphate reductase assembly flavoprotein NrdI	gp84	gp87	\	\	\
ribonucleotide reductase subunit alpha(NrdE)	gp85	gp86	\	\	\
class 1b ribonucleoside-diphosphate reductase subunit beta(NrdF)	gp86	gp85	\	\	\
thymidylate synthase	gp100	gp55	\	\	\
Dihydrofolate reductase (DHFR)	\	gp52	\	\	\
Deoxycytidylate deaminase (EC 3.5.4.12)	gp125	\	\	\	\

## Data Availability

The original contributions presented in this study are included in the article. Further inquiries can be directed to the corresponding authors.
